# Primary malignant melanoma of the uterine cervix: a case report of aggressive progression despite multimodal therapy

**DOI:** 10.3389/fonc.2026.1803729

**Published:** 2026-03-25

**Authors:** Qing Yu

**Affiliations:** Department of Pathology, Deyang People’s Hospital, Deyang, China

**Keywords:** case report, cervical melanoma, immunotherapy resistance, mucosal melanoma, NRAS mutation, targeted therapy

## Abstract

**Objective:**

Primary malignant melanoma of the uterine cervix is an exceptionally rare malignancy with a biology distinct from its cutaneous counterpart and a dire prognosis. This case provides a critical *in vivo* model for interrogating the therapeutic resistance mechanisms inherent to mucosal melanomas.

**Methods:**

We report a case of a 73-year-old woman with early-stage (pT1aN0), NRAS Q61K-mutant primary cervical melanoma.

**Results:**

The patient progressed rapidly through radical surgery and adjuvant Toripalimab (anti-PD-1) immunotherapy. Despite salvage therapy with a MEK inhibitor, she succumbed to the disease 17 months after diagnosis. The sequential failure of both immune checkpoint blockade and targeted pathway inhibition highlights a dual-layer of resistance.

**Conclusions:**

This experience mandates a fundamental re-evaluation of adjuvant strategies for this disease. It provides a compelling rationale for upfront, biology-driven combination trials (e.g., immunotherapy plus MEK or CDK4/6 inhibition) in the neoadjuvant or adjuvant setting for high-risk cases.

## 1 Introduction

Malignant melanoma (MM) primarily originates from cutaneous melanocytes but can arise in mucosal membranes, constituting a distinct and more aggressive subtype. Primary melanoma of the uterine cervix is extraordinarily rare, accounting for less than 2% of all cervical malignancies (1) and posing significant diagnostic and therapeutic challenges (2). Patients typically present with nonspecific symptoms such as postmenopausal bleeding, often leading to diagnostic delays. The tumor’s biology is notoriously aggressive, with a high propensity for local recurrence and early distant metastasis. Current treatment strategies are largely extrapolated from experience with cutaneous melanoma and other mucosal sites (3), lacking robust evidence specific to the cervical origin (4). This case report details the comprehensive management and ultimately fatal trajectory of a patient with primary cervical melanoma. It vividly illustrates the limitations of existing therapeutic modalities and underscores the urgent need for biology-driven, novel treatment approaches tailored to this unique disease.

## 2 Case presentation

### 2.1 Clinical presentation and history

A 73-year-old, G2P2, postmenopausal woman presented in June 2024 with a 4-month history of unexplained vaginal bleeding. Her past medical history was significant for a 30+ year history of hypertension, with a maximum recorded blood pressure of 200/110+ mmHg. This was managed with long-term oral Indapamide (2.5 mg qn) and Nifedipine sustained-release tablets (20 mg every morning, 10 mg every night). There was no history of other chronic illnesses, prior surgeries, blood transfusions, or allergies. Family history was non-contributory to any malignancies. Psychosocially, the patient lived with her family and initially demonstrated a strong support system.

Gynecological examination revealed a dark red, soft, pedunculated mass (approximately 2×2 cm) on the posterior vaginal wall at the 7 o’clock position, with a correlating smaller, light-brown lesion on the cervix.

### 2.2 Diagnostic workup and pathological confirmation

**Figure 1:**
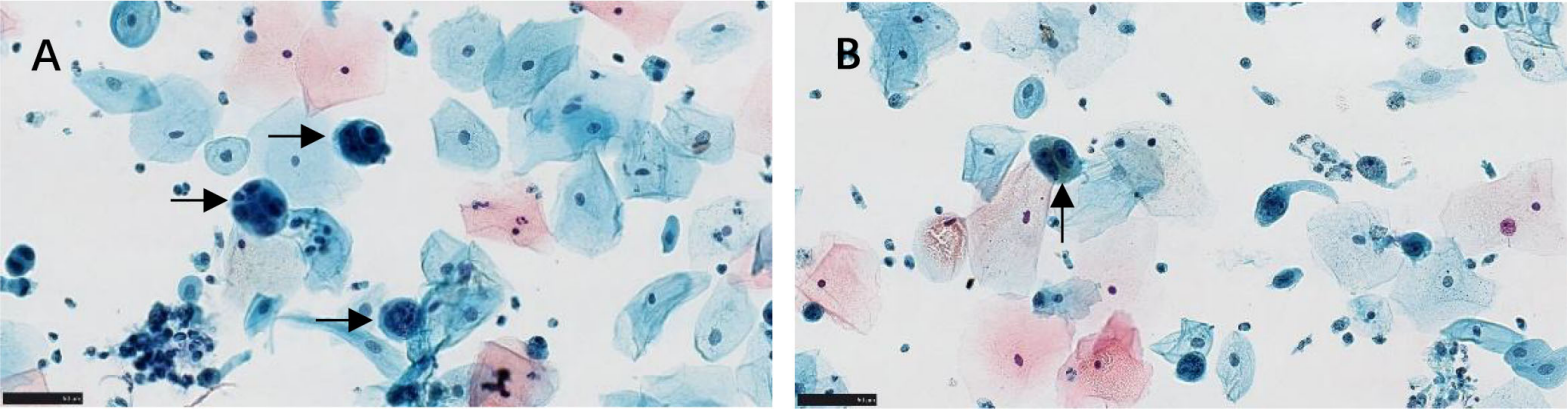
Diagnostic cytology (ThinPrep). **(A)** Atypical cells with high nuclear-to-cytoplasmic ratios and hyperchromasia (arrow). **(B)** Malignant cell with fine intracytoplasmic melanin pigment (arrowhead). Scale bars: 50 µm.

Initial investigation included a cervical liquid-based cytology (ThinPrep) test. Cytological examination revealed scattered atypical cells characterized by high nuclear-to-cytoplasmic ratios and occasional fine cytoplasmic melanin pigments (**Figure 1**), findings highly suggestive of a malignant melanocytic lesion. This prompted a targeted biopsy of the vaginal mass.

**Figure 2:**
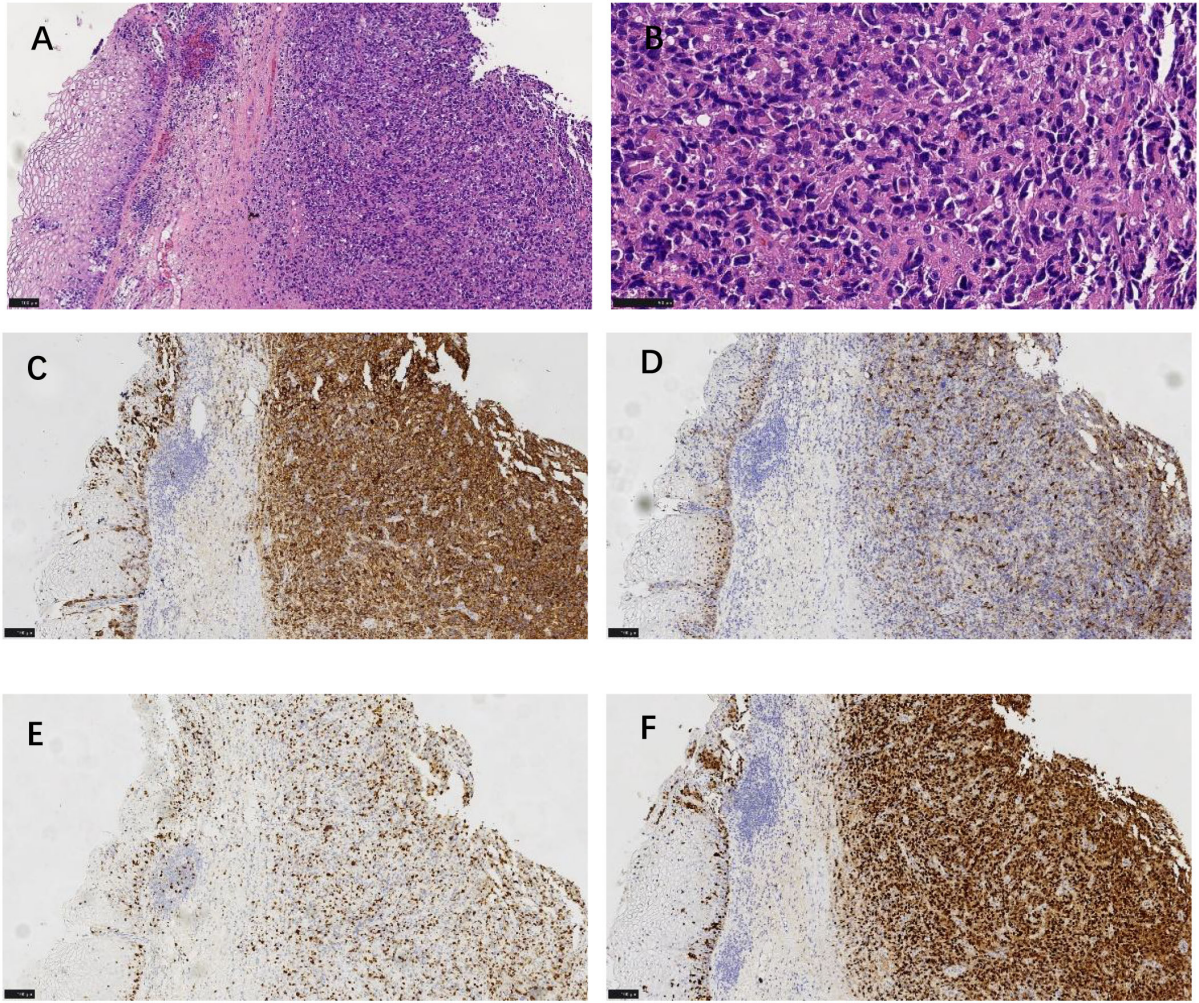
Histopathology and immunohistochemistry. **(A)** H&E, low power. **(B)** H&E, high power showing epithelioid cells with melanin pigment. **(C)** S-100: diffuse positivity. **(D)** HMB45: diffuse positivity. **(E)** Ki-67: high proliferative index (~60%). **(F)** SOX-10: strong nuclear positivity. Scale bars: 100 µm **(A, C-F)**; 50 µm **(B)**.

Histopathological analysis of the biopsy confirmed a malignant neoplasm composed of epithelioid and spindle cells featuring prominent nucleoli and abundant intracytoplasmic melanin pigment (**Figures 2A, B**). Immunohistochemistry (IHC) profiling demonstrated that the tumor cells were diffusely and strongly positive for melanocytic markers S-100 (**Figure 2C**), HMB45 (**Figure 2D**), Melan-A, and SOX-10 (**Figure 2F**), and were positive for vimentin. A remarkably high Ki-67 proliferation index of 60% was noted (**Figure 2E**). Epithelial (Pan-CK, EMA, CK5/6), squamous (P40, P63), hormonal (ER, PR), and lymphoid (LCA) markers were uniformly negative (**Table 1**). This immunoprofile definitively established the diagnosis of malignant melanoma.

**Table 1:** Initial diagnostic immunohistochemical profile (vaginal biopsy).

Antibody	Result	Lineage/Comment
S-100	Positive (+)	Melanocytic/Neural
HMB45	Positive (+)	Melanocytic
Melan-A (MART-1)	Positive (+)	Melanocytic
SOX-10	Positive (+)	Melanocytic/Neural Crest
Ki-67	Positive (60%)	High proliferation index
Vimentin	Positive (+)	Mesenchymal
P53	Positive (+)	Mutant pattern (overexpression)
Pan-CK (AE1/AE3)	Negative (-)	Epithelial
EMA	Negative (-)	Epithelial
CK5/6	Negative (-)	Squamous/Basal cell
P40	Negative (-)	Squamous cell carcinoma
P63	Negative (-)	Squamous/Myoepithelial
ER	Negative (-)	Hormone receptor
PR	Negative (-)	Hormone receptor
NapsinA	Negative (-)	Pulmonary/renal adenocarcinoma
WT1	Negative (-)	Mesothelial/ovarian serous carcinoma
LCA (CD45)	Negative (-)	Hematolymphoid

Subsequent whole-body 18F-FDG PET/CT scan (July 2024) revealed focal hypermetabolism confined to the primary vaginal/cervical site, with no evidence of locoregional or distant metastasis. The diagnosis was corroborated by an external pathology review at a tertiary cancer center.

Notably, the vaginal lesion was completely excised during the initial local excision procedure in July 2024, while the cervical lesion persisted and was confirmed in the subsequent radical hysterectomy specimen. This temporal sequence—resolution of the vaginal lesion after local excision versus persistence of the cervical lesion—supports the interpretation of the cervix as the primary site, with the vaginal lesion representing either a satellite or a metastatic deposit.

### 2.3 Initial and radical surgical management

The patient underwent initial excision of the vaginal and cervical lesions in July 2024. Pathological examination confirmed complete removal of the vaginal lesion with negative margins. Following pathological confirmation, definitive surgical management was pursued via a radical laparoscopic hysterectomy with bilateral salpingo-oophorectomy and pelvic lymphadenectomy in August 2024.

Pathological examination of the hysterectomy specimen confirmed mucosal melanoma of the cervix, with a maximum invasion depth of 2 mm. Of note, no residual tumor was identified at the prior vaginal surgical site, confirming complete excision of the vaginal lesion. All surgical margins and resected pelvic lymph nodes (0/15) were negative for malignancy, establishing a final pathological stage of pT1aN0 (FIGO Stage IA).

### 2.4 Adjuvant therapy and surveillance

Tumor genetic profiling identified an NRAS Q61K mutation. Given this high-risk molecular feature, adjuvant systemic therapy was recommended. The patient opted for immunotherapy and subsequently received adjuvant Toripalimab (anti-PD-1, 160 mg IV every two weeks) for approximately 11 months, which was well-tolerated.

### 2.5 Disease recurrence and salvage therapy

In July 2025 (11 months post-diagnosis), surveillance imaging revealed pelvic soft tissue recurrence, metastatic involvement of para-aortic and pelvic lymph nodes, and an iliac bone metastasis. A multimodal salvage regimen was initiated:

1. Palliative Radiotherapy: A total dose of 60 Gy in 30 fractions was delivered to the pelvic mass and iliac bone metastasis (September to November 2025).
2. Bone-Targeted Therapy: Denosumab (120 mg SC every 4 weeks) was administered.
3. Targeted Therapy: Based on the NRAS Q61K mutation, the patient was commenced on Tolomeitinib (妥拉美替尼), a highly selective MEK1/2 inhibitor. It is important to clarify that Tolomeitinib is an investigational agent approved by the National Medical Products Administration (NMPA) of China for the treatment of NRAS-mutant melanoma. In this case, it was administered as salvage therapy following disease progression on immunotherapy. Unfortunately, no re-biopsy of the progressing lesion was performed at the time of resistance to investigate potential acquired resistance mechanisms, such as MAPK pathway reactivation or secondary mutations. This represents a limitation of the study.During radiotherapy, the patient developed manageable Grade 2 radiation proctitis, treated with supportive care.

### 2.6 Final outcome

Despite aggressive salvage therapy, the patient’s condition deteriorated with systemic progression. She died on November 30, 2025, approximately 17 months after the initial diagnosis. The clinical timeline is summarized in **Table 2**.

**Table 2:** Timeline of key clinical events and interventions.

Date/Period	Category	Event and description	Key outcome/Notes
Jun 2024	Diagnostic Work-up	Initial Screening. Cervical liquid-based cytology (ThinPrep) performed for postmenopausal bleeding.	Cytolog**y:** Revealed atypical cells with high N/C ratio and cytoplasmic melanin pigment, raising suspicion for a melanocytic lesion.
Jun 2024	Diagnosis	Definitive Diagnosis. Biopsy of vaginal mass with immunohistochemistry (IHC).	IHC Profile: S-100+, HMB45+, Melan-A+, SOX-10+, Ki-67 60%. Diagnosis: Malignant melanoma.
10 Jul 2024	Surgery	Initial Local Excision. Excision of vaginal and cervical lesions.	Pathology confirmed melanoma.
29 Jul 2024	Staging	Systemic Staging. PET/CT scan at a tertiary center.	Localized disease: Focal hypermetabolism at primary site; no distant metastasis.
16 Aug 2024	Surgery	Radical Surgery. Laparoscopic radical hysterectomy, bilateral salpingo-oophorectomy, and pelvic lymphadenectomy.	Final Pathologic Stage: pT1aN0 (FIGO Stage IA). All margins and lymph nodes (0/15) negative.
Aug 2024	Biomarker	Genetic Testing Result.	NRAS mutation identified.
Sep 2024 – Jul 2025	Medical Therapy (Adjuvant)	Adjuvant Immunotherapy. Toripalimab (anti-PD-1), 160 mg IV every two weeks.	Initiated based on high-risk features. Duration ~11 months.
Jul 2025	Disease Progression	Recurrence and Metastasis. Imaging confirmed pelvic recurrence, nodal and bone (iliac) metastases.	Progressive disease occurred during adjuvant therapy.
19 Sep – 04 Nov 2025	Radiotherapy	Palliative Radiotherapy. To pelvic mass and bone metastasis. Total dose: 60 Gy/30f.	Complicated by manageable grade 2 radiation proctitis.
Starting Sep 2025	Medical Therapy (Supportive)	Bone-Targeted Therapy. Denosumab, 120 mg SC every 4 weeks.	For prevention of skeletal-related events.
Nov 2025	Medical Therapy (Targeted)	Targeted Therapy. Oral Tolomeitinib (妥拉美替尼,MEK inhibitor) initiated.	Salvage therapy based on NRAS mutation. **Investigational agent used (NMPA-approved); no re-biopsy performed at resistance.**
30 Nov 2025	Outcome	Patient Death. Due to disease progression with systemic symptoms.	Overall Survival (OS): ~17 months.

Bold text indicates a key limitation of this study: no re-biopsy was performed at the time of disease progression to investigate potential acquired resistance mechanisms. Tolometinib is an investigational MEK inhibitor approved by China's National Medical Products Administration (NMPA) for NRAS-mutant melanoma.

### 2.7 Patient perspective

According to her family and clinical records, the patient initially maintained a hopeful outlook and was motivated to pursue aggressive treatment after the initial diagnosis. She actively participated in decision-making and consented to radical surgery and adjuvant immunotherapy. However, the discovery of disease recurrence in July 2025 led to a significant decline in her morale. A poignant reflection of this psychological shift was her decision to decline immediate hospitalization for salvage therapy, opting instead for traditional Chinese medicine for a period, which temporarily delayed the initiation of radiotherapy and targeted therapy. This decision underscores the profound emotional toll and the complex decision-making process patients face when confronting a lethal disease with a poor prognosis. During subsequent salvage radiotherapy, she frequently reported fatigue and pain, which substantially diminished her quality of life.

## 3 Discussion

This case documents a sobering clinical course that underscores primary cervical melanoma’s aggressive biology, demonstrating the stark limitations of current therapeutic paradigms even when applied sequentially and with precision. It underscores that diagnostic precision does not guarantee therapeutic efficacy for malignancies with innate virulence, compelling a fundamental re-evaluation of our approaches.

### 3.1 Diagnostic vigilance: the role of cytology and histology

The initial cytology, by identifying atypical cells with cytoplasmic melanin, played a crucial triggering role. The diagnostic cornerstone remains histopathology with a comprehensive IHC panel. The co-expression of S-100, HMB45, Melan-A, and SOX-10, alongside a high Ki-67 index (60%), was classic for melanoma and prognosticated aggressive behavior.

### 3.2 Navigating therapeutic challenges

Surgery as the Foundation: Radical surgery achieved local control and accurate staging (pT1aN0), remaining the primary modality for potential cure in localized disease.

The Adjuvant Immunotherapy Conundrum: The use of adjuvant Toripalimab reflects the extrapolation of cutaneous melanoma data. The 11-month disease-free interval may signify transient benefit, but ultimate progression highlights the issue of innate or acquired resistance to immune checkpoint inhibitors in the immunosuppressive microenvironment of mucosal melanomas (5).

The Unanswered Question of Adjuvant Radiotherapy: The pattern of recurrence in this patient, with a significant component of pelvic failure, raises the pertinent question of whether adjuvant pelvic radiotherapy could have played a role. In retrospect, the use of radiation in the adjuvant setting might have offered several theoretical advantages. Firstly, it could have provided enhanced locoregional control, potentially delaying or preventing the pelvic soft tissue and nodal recurrence observed. Secondly, there is a growing body of evidence suggesting a synergistic effect between radiotherapy and immunotherapy, where radiation-induced immunogenic cell death can convert the tumor into an *in-situ* vaccine, potentially augmenting the systemic anti-tumor immune response—a phenomenon known as the abscopal effect (6). This synergy is particularly relevant for “cold” tumors like mucosal melanoma, which are notoriously resistant to checkpoint inhibitors alone.

At the time of our patient’s treatment, however, the role of adjuvant radiotherapy for stage IA cervical melanoma was not clearly defined, and current paradigms for early-stage, completely resected disease often prioritize systemic therapy over local radiation to address the high risk of distant micrometastasis. Concerns regarding potential added toxicity, particularly in the pelvic region, also factored into the decision-making process. This case, with its unfortunate pelvic component of failure, serves to highlight this therapeutic dilemma and suggests that the potential benefit of combining radiotherapy with immunotherapy in the adjuvant setting for high-risk mucosal melanomas warrants prospective investigation, as advocated in the recent literature (6).

Precision Palliation in Metastatic Disease: The employed multimodal salvage regimen exemplifies modern palliative oncology. However, the limited efficacy of single-agent MEK inhibition observed here (7) underscores the need for more potent combinatorial strategies (e.g., MEK + CDK4/6 inhibition (8)).

The Prognostic Implications of NRAS: While providing an actionable target, the NRAS mutation in mucosal melanoma is often associated with a worse prognosis (1, 9), compounding the therapeutic challenge.

### 3.3 Determining the primary site: evidence supporting cervical origin

A key consideration raised during the review process was the definitive assignment of the primary site, given the presence of synchronous melanocytic lesions in both the cervix and vagina at initial presentation. The diagnostic gold standard for mucosal melanoma—identification of an *in-situ* (junctional) component—was not definitively documented in the cervical lesion. However, the clinical course provides compelling indirect evidence supporting a cervical primary.

Crucially, the vaginal lesion was completely excised during the initial local procedure in July 2024, with histologically confirmed negative margins. In contrast, the cervical lesion persisted and was confirmed in the subsequent radical hysterectomy specimen (August 2024). If the vagina were the primary site, complete excision of the vaginal primary would be expected to eliminate the source of any metastatic deposits; yet the cervical lesion remained. This temporal sequence—resolution of the vaginal lesion after local excision versus persistence of the cervical lesion—strongly supports the interpretation of the cervix as the primary site, with the vaginal lesion representing either a synchronous satellite lesion or a metastatic deposit from the cervical primary.

While we acknowledge the absence of a documented junctional component as a limitation, the clinical and surgical course provides robust circumstantial evidence for cervical origin. We have therefore maintained the designation of primary cervical melanoma in the title and throughout the manuscript.

### 3.4 Translational implications and future directions

The clinical progression suggests concurrent resistance mechanisms: an immunosuppressive “cold” tumor microenvironment limiting immunotherapy efficacy (5), followed by rapid bypass signaling upon MEK inhibition (9). This indicates that effective therapy may require concurrent dual-pathway targeting from the outset.

Preclinical models show that combined MEK and CDK4/6 inhibition can induce sustained responses in NRAS-mutant melanoma (8, 9). Our clinical experience provides human validation for these findings and urgently supports translating such rational combinations into clinical trials.

Therefore, future paradigms should test frontline combination strategies. The neoadjuvant setting offers a powerful window to assess biologic activity and resistance mechanisms early. Centralized referral of these rare cases to centers capable of executing biomarker-driven trials is essential (10).

To translate this centralized approach into actionable progress, we propose that future clinical trials for NRAS-mutant mucosal melanomas should prioritize upfront combination strategies. Specifically, Phase I/II trials evaluating neoadjuvant or adjuvant regimens combining immune checkpoint inhibitors (e.g., anti-PD-1) with MEK inhibitors and/or CDK4/6 inhibitors are warranted, accompanied by robust correlative biomarker studies to identify predictors of response and mechanisms of resistance.

## 4 Conclusion

This case report starkly illustrates the aggressive biology of primary cervical melanoma. Despite early-stage diagnosis and access to contemporary multimodal therapy, the disease followed a rapidly fatal course. This outcome compels a dual paradigm shift: from reactive intervention to proactive, biology-driven strategy, and from late-stage palliation to its early integration within the treatment framework. Future efforts must prioritize centralized expertise (10), biology-driven clinical trials testing novel combinations (e.g., immunotherapy + targeted pathway inhibition) (8), and sustained translational research into the unique biology of mucosal melanomas (5, 9). Only through such a concerted effort can we hope to improve outcomes for this lethal disease.

## Data Availability

The original contributions presented in the study are included in the article/Supplementary Material. Further inquiries can be directed to the corresponding author.
